# Investigating antimicrobial features and drug interactions of sedoanalgesics in intensive care unit: an experimental study

**DOI:** 10.5599/admet.1042

**Published:** 2021-09-06

**Authors:** Ozge Unlu, Emre Sertac Bingul, Sevgi Kesici, Mehmet Demirci

**Affiliations:** 1 Istanbul Atlas University, Faculty of Medicine, Department of Medical Microbiology, Turkey; 2 Recep Tayyip Erdogan University, Training and Research Hospital, Department of Anesthesiology and Reanimation, Turkey; 3 Health Sciences University, Hqamidiye Etfal Training and Research Hospital, Department of Anesthesiology and Reanimation, Turkey; 4 Kirklareli University, Faculty of Medicine, Department of Medical Microbiology, Turkey

**Keywords:** Sedoanalgesics, nosocomial agents, antimicrobial effects, drug interactions

## Abstract

**Study Objective:**

Aim of this study was to evaluate antimicrobial effects and interaction between analgesic combinations of fentanyl citrate, dexmedetomidine hydrochloride and tramadol hydrochloride on *Staphylococcus aureus, Klebsiella pneumoniae, Escherichia coli, Pseudomonas aeruginosa* and *Candida albicans* which are some of the most common nosocomial infection related microorganisms.

**Design:**

In vitro prospective study.

**Setting:**

University Clinical Microbiology Laboratory.

**Measurements:**

In order to evaluate in vitro antimicrobial effects and interaction between analgesic combinations, tramadol hydrochloride, fentanyl citrate and dexmedetomidin were used against *S. aureus* ATCC 29213, *K. pneumoniae, E. coli* ATCC 25922, *P. aeruginosa* ATCC 27853 and *C. albicans* ATCC 10231 standard strains by microdilution method.

**Main Results:**

According to microdilution assays tramadol has shown the most efficient antimicrobial activity also it has been observed that 10 μg/ml concentrated dexmedetomidine has antimicrobial effects on *S. aureus, K. pneumoniae* and *P. aeruginosa*. Fentanyl has displayed evident inhibitory potency on the pathogens except for *Klebsiella pneumoniae*, nevertheless our predefined minimum concentration inhibited growth by 9.5 %. Fentanyl and dexmedetomidine together exhibited more antimicrobial effect on *P. aeruginosa* and *E. coli* growth. Additionally, when the three drugs examined together, microbial inhibition occurred more than expected on *E. coli* again and also on *C. albicans* growth.

**Conclusions:**

Our results revealed the antimicrobial properties and synergy with the different combinations of fentanyl, dexmedetomidine and tramadol against the most common nosocomial infection agents in the ICU. This is the first study in the literature looking into the microbial “interactions” of opioids and sedative drugs but more research is needed in order to define clinico-laboratory correlation.

## Introduction

Intensive care patients often require sedation procedures for several reasons during their hospitalization. Since intensive care units (ICU) are prone to nosocomial infections; intravenous (IV) medications gain more importance in the aspect of microbial features. Contamination based infections may complicate clinical follow-up and little is known whether the sedative agents have antimicrobial properties. Among them, propofol is a well-studied drug that is now known to be inducing bacterial growth [1–4]. However, there are many other drug combinations used in routine practice to maintain sedation.

α-2 selective blocker dexmedetomidine has become more popular and widely used for its less respiratory depressant effects instead of midazolam which triggers delirium in the elderly [5–7]. Many clinicians also add opioids to the treatment just to deepen sedation and fentanyl appears to be one of the most common choices due to its hemodynamic stable properties. There are studies comparing dexmedetomidine and midazolam from the microbiological aspect in the literature [8,9]. But since midazolam has gained a bad reputation for delirium triggering properties another sedative agent fentanyl is yet to be explored.

This study aimed to evaluate antimicrobial effects and interaction between analgesic combinations of fentanyl citrate, dexmedetomidine hydrochloride and tramadol hydrochloride on *Staphylococcus aureus, Klebsiella pneumoniae, Escherichia coli, Pseudomonas aeruginosa* and *Candida albicans* which are some of the most common nosocomial infection related microorganisms. These drugs were chosen for being the most frequently used infusion sedoanalgesics in our institute.

## Materials and methods

### Tested sedoanalgesics

Tramadol hydrochloride (ultramex), fentanyl citrate (talinat) and dexmedetomidin (hipnodex) were tested to reveal their antimicrobial activity and interaction between analgesic combinations.

### Bacterial strains

*S. aureus* ATCC 29213, *K. pneumoniae, E. coli* ATCC 25922, *P. aeruginosa* ATCC 27853 and *C. albicans* ATCC 10231 standard strains, which are primary pathogens of bacteremia and sepsis were chosen in order to compare analgesics antimicrobial activity.

### Antibacterial broth test

Antimicrobial activity of the selected analgesics was measured with microdilution method according to the instructions of Clinical Laboratory Standards Institute (CLSI). Tramadol hydrochloride (Abbott, Rocky Mount, NC, USA, supplied in a 2 ml ampoule containing 50 μg/ml in sodium chloride solution) was diluted with Brain Heart Infusion Broth (BHI) to the final concentrations of 500 μg/ml, 250 μg/ml, 125 μg/ml, 62.5 μg/ml, 31.25 μg/ml. Fentanyl citrate (Abbott, Rocky Mount, NC, USA, supplied in a 10 ml ampoule containing 0,05 mg/ml in sodium chloride solution) was diluted with Brain Heart Infusion Broth (BHI) to the final concentrations of 5 μg/ml, 2.5 μg/ml, 1.25 μg/ml, 0.625 μg/ml, 0.3125 μg/ml. Dexmedetomidine hydrochloride (Abbott, Rocky Mount, NC, USA, supplied in a 2 ml ampoule containing 100 μg/ml in sodium chloride solution) was diluted with Brain Heart Infusion Broth (BHI) to the final concentrations of 10 μg/ml, 5 μg/ml, 2.5 μg/ml, 1.25 μg/ml, 0.625 μg/ml. Overnight broth cultures of *S. aureus* ATCC 29213, *K. pneumoniae ATCC 13883, E. coli* ATCC 25922, *P. aeruginosa* ATCC 27853 and *C. albicans* ATCC 10231 were adjusted to the turbidity of a 0.5 McFarland standard. 10 μl of each strain were inoculated to each well. Broths without any analgesic materials were served as controls for comparison. Plates incubated overnight and bacterial growth in each well were measured with Epoch spectrophotometer (Biotek, Germany) at 600 nm.

### Interaction between analgesic combinations via broth microdilution method

Tramadol hydrochloride (TH), fentanyl citrate (FC) and dexmedetomidine hydrochloride (DH) combinations were diluted in Brain Heart Infusion Broth (BHI) with or without *S. aureus* ATCC 29213, *K. pneumoniae ATCC 13883, E. coli* ATCC 25922, *P. aeruginosa* ATCC 27853 and *C. albicans* ATCC 10231 were adjusted to the turbidity of a 0.5 McFarland standard. 10 μl of each strain were inoculated to each well. Broths without any analgesic materials were served as controls for comparison. Plates were incubated overnight and bacterial growth in each well were measured with Epoch spectrophotometer (Biotek, Germany) at 600 nm. The preparation of the microdilution in microwells with single agent concentrations of the analgesic to determine the minimum inhibitory concentration (MIC), control wells and paired combinations of the analgesics were used in unique concentrations to determine the fractional inhibitory concentration (FIC). The value of the ∑FIC index is then used to determine whether synergism, indifference or antagonism occurred between the antimicrobial agents and it was used to interpret the nature of the interactions: synergism ≤0.5, indifference >0.5 to ≤4, antagonism >4 according to American Society of Microbiology [10].

## Results

### Antimicrobial activity for analgesics

Antimicrobial susceptibility results revealed that 500 μg/ml tramadol hydrochloride inhibited the growth of *K. pneumoniae* 100 %. Also, inhibition rates for *P. aeruginosa*, *S. aureus, E. coli*, and *C. albicans* were 40, 35, 28.8 and 37.5 %, respectively. Moreover, the inhibitory effects of tramadol hydrochloride were observed at descending rates until the lowest concentration tested which was 31,25 μg/ml for *K. pneumoniae, E. coli*, and *C. albicans*.

In addition, 5 μg/ml fentanyl citrate inhibited the growth of *S. aureus, P. aeruginosa*, *E. coli*, and *C. albicans* at the following rates 38, 13.3, 4.5 and 7.5 %, respectively. Also, fentanyl citrate did not show any inhibitory effect on the growth of *K. pneumoniae* at maximum concentration tested, whereas it inhibited growth 9.5 % in its minimum concentration (3,125 μg/ml).

Moreover, 10 μg/ml dexmedetomidine had an inhibitory effect on the growth of *S. aureus, K. pneumoniae* and *P. aeruginosa* at the rates of 34, 21 and 12.1 %, respectively. Also, dexmedetomidine did not show any inhibitory effect on the growth of *E.coli* at maximum concentration tested, whereas it inhibited growth 5 % in its minimum concentration. Moreover, it has been observed that the growth of *C. albicans* inhibited 8.4 % at the maximum concentration of the analgesic while it has been inhibited 28 % at the minimum concentration tested.

All the results of microdilution assays for tramadol hydrochloride, fentanyl citrate and dexmedetomidine were given in Table 1, Table 2 and Table 3, respectively.

According to microdilution results, tramadol hydrochloride had the most powerful effect against bacterial growth. Moreover, antimicrobial susceptibility results suggest that antimicrobial effect of analgesics vary according to the type of the analgesic, pathogen and the concentration of the material. Also, higher concentration does not always result higher antimicrobial effect.

### Interaction between analgesic combinations

∑FIC index for tramadol hydrochloride, fentanyl citrate and dexmedetomidine hydrochloride combinations against standard strains were shown in Table 4.

When the interactions between analgesic materials were analyzed, it was found that FC + DH combination had a synergistic antimicrobial effect on the growth of *E. coli* and *P. aeruginosa* standard strains. Moreover, it has been observed that TH + FC + DH combination had a synergistic antimicrobial effect on *E. coli* and *C. albicans* standard strains.

## Discussion

In this study, we have observed that 10 μg/ml concentrated dexmedetomidine has antimicrobial effects on *S.aureus*, *K. pneumoniae* and *P. aeruginosa*. These findings were slightly different from a previous study in which researchers have established the same activity with higher (32 μg/ml) concentrations [10]. Even though dexmedetomidine is a more preferred sedative drug than midazolam recently; antimicrobial effects were not described as well as it has been studied for midazolam. Dexmedetomidine seems to inhibit bacterial growth when prepared in correct concentrations. Commonly used infusions are prepared at a concentration of 4 μg/ml and according to our results, 10 μg/ml infusion fluids might be sufficient enough to prevent bacterial colonization.

One should declare that Keles *et al*. [9] have not found any antimicrobial effect of dexmedetomidine. However, this difference may be caused by the chosen microbiological assays. We have performed spectrophotometry after microdilution in BHI which is more sensitive than incubation in blood agar that Keles and her colleagues chose.

Dexmedetomidine hydrochloride does not contain preservatives or stabilizers. Therefore the inhibitor effect might be related to its chemical structure. The actual mechanism is unclear and also no microbial growth due to dexmedetomidine was declared in the literature until now [8,9]. In addition to its antimicrobial effect, Taniguchi *et al*. [11] have demonstrated anti-inflammatory features of this drug in rats. According to this study, α2-adrenoceptor agonist has modulated interleukin and TNF-α responses reducing excessive neutrophil accumulation on lung tissue. With that knowledge, dexmedetomidine might be a more appropriate drug than midazolam in septic patients who are in need of analgesia and sedation which requires further clinical studies.

Fentanyl citrate has displayed evident inhibitory potency on the pathogens except for *Klebsiella pneumoniae*, nevertheless our predefined minimum concentration inhibited growth by 9.5 %. To our knowledge, there are few studies focused on fentanyl’s antimicrobial properties and some of them oppose our results [12]. For instance; Rota *et al*. [13] were not able to demonstrate the inhibiting effect of fentanyl. In contrast, Graystone *et al*. [14] have investigated 15 medications that are used in ICU and concluded all of them except propofol were bactericidal including fentanyl. Based on spectrophotometric calculations our study method was not designed to investigate the bactericidal effects of the drugs. However, as interpreted in the mentioned studies, we may propose specific assumptions. Fentanyl citrate ampoules contain 500 μg/10 ml which is a large dose for one use only. Therefore, multi-dosing with the same syringe is a quite common application practice in the ICU settings [12-15]. It could be argued that the drug would degrade and become susceptible to contamination during the time kept at room temperature in the same syringe. However, fentanyl is a physicochemically stable drug and can be stored in the syringe up to 1 week as a mixture with other drugs such as midazolam under 32 °C [16]. Refilling of the syringes with this drug does not cause microbial contamination as observed in one prospective longitudinal study [12]. The reliability of multi-dosing with the same syringe is often questioned by the clinicians and considering our results this approach seems adequate. Perhaps further clinical studies are required.

A fentanyl analog “remifentanil” was investigated by Apan *et al*. [17] in the same manner. They have observed similar microbial inhibition on *S. aureus*, *P. aeruginosa*, *E. coli* and *C. albicans* depending on the concentration. However, this effect was attributed to “glycine” which is present in remifentanil ampoules as a preservative. It might be possible that glycine has bacteriostatic potency but since both remifentanil and fentanyl are 4-anilidopiperidine structured drugs, further evaluations are needed including also alfentanil and sufentanil [18,19]. Fentanyl citrate does not contain preservatives.

Tramadol is known to be a “good” analgesic that has few respiratory and hemodynamic adverse effects [20]. Despite being a weak opioid analgesic, it provides adequate analgesia especially in elder patients who are prone to cognitive dysfunction [21]. Therefore, it is mostly used for the postoperative period especially after orthopedic surgery in intensive care units. Many clinicians do not reckon tramadol as a sedative agent. But when combined with dexmedetomidine, these drugs show remarkable synergistic effects [20]. As a striking result of our study tramadol has shown the most efficient antimicrobial activity. From this aspect preparing combined infusions of dexmedetomidine and tramadol is rather considerable due to their fewer side-effect properties. It is worth noting that the synergy occurs only on sedation and analgesia but not on bacteriostatic activity. To the best of our knowledge, this is the only study examining antimicrobial features of tramadol.

Intravenous treatments are generally applied via central venous catheters in the ICUs. Occasionally multiple sedative drugs are needed to be given from the same catheter line and usually infused at lower rates. Whether these drug combinations covering the same line attenuate catheter related nosocomial infections is questionable. We have observed evident synergy with the different combinations against particular microorganisms in our study. Fentanyl and dexmedetomidine together exhibited more antimicrobial effects on *P. aeruginosa* and *E. coli* growth. Additionally, when the three drugs were examined together, microbial inhibition occurred more than expected on *E. coli* again and also on *C. albicans* growth. This interaction needs to be evaluated more. Since this is an *in vitro* study, *in vivo* examinations are necessary to adapt clinical practice. As far as we know there is no other study in the literature looking into the microbial “interactions” of opioids and sedative drugs. We believe these interactions are remarkably important which may affect routine IV therapy and shape the practitioner’s approach. As a well-known fact, critical patients receiving total parenteral nutrition via the central venous catheters are at great risk for *candidemia*. The combination of TH, FC and DH infusion is worth investigating from this aspect. It is difficult to come to a conclusion with our laboratory results for sure but further *in vitro* and *in vivo* studies may reveal clinically significant interactions.

Perhaps the most important limitation of our study was the lack of hourly made spectrophotometric calculations. Therefore we could not demonstrate microbial inhibition or growth trend during 24 hours. Interestingly, Graystone *et al*. [14] have observed an increase in *S. aureus* number during the first 4 hours which declined later with all opioids except fentanyl. Pyrogenic reactions are reported because of extrinsically contaminated fluids even if there is no bacteremia [22]. Bacterial load in infusion fluids may be reduced by choosing the appropriate agent and concentration to enhance patient comfort in both sedation and anti-inflammatory meanings.

## Conclusions

We have observed definite antimicrobial properties and synergy with the different combinations of fentanyl, dexmedetomidine and tramadol against the most common nosocomial infection agents in the ICU. This is the first study in the literature looking into the microbial “interactions” of opioids and sedative drugs but more research is needed in order to define clinico-laboratory correlation.

Limitations of this work are: (i) the study is in vitro study and (ii) clinical practice is required.

## Figures and Tables

**Table 1.** Antimicrobial effects of tramadol hydrochloride in different concentrations against to tested microorganisms

Microorganisms	500 μg/ml	250 μg/ml	125 μg/ml	62.5 μg/ml	31.25 μg/ml
***K. pneumoniae***	100.00 %	80.02 %	58.41 %	58.06 %	60.63 %
***P. aeruginosa***	40.03 %	0.00 %	0.00 %	0.00 %	0.00 %
***E. coli***	28.74 %	28.74 %	20.49 %	7.77 %	3.89 %
***S. aureus***	34.62 %	33.46 %	26.25 %	7.21 %	0.00 %
***C. albicans***	35.55 %	26.39 %	27.47 %	27.11 %	27.65 %

**Table 2.** Antimicrobial effects of fentanyl citrate in different concentrations against to tested microorganisms

Microorganisms	5 μg/ml	2.5 μg/ml	1.25 μg/ml	0.625 μg/ml	0.3125 μg/ml
***K. pneumoniae***	0.00 %	4.09 %	6.78 %	6.89 %	9.58 %
***P. aeruginosa***	13.34 %	8.67 %	4.85 %	1.04 %	0.69 %
***E. coli***	4.59 %	6.60 %	6.95 %	6.48 %	7.77 %
***S. aureus***	38.22 %	39.64 %	34.75 %	21.24 %	1.93 %
***C. albicans***	7.54 %	26.57 %	36.45 %	38.24 %	30.70 %

**Table 3.** Antimicrobial effects of dexmedetomidine in different concentrations against to tested microorganisms

Microorganisms	10 μg/ml	5 μg/ml	2.5 μg/ml	1.25 μg/ml	0.625 μg/ml
***K. pneumoniae***	21.14 %	8.53 %	3.04 %	0.00 %	0.00 %
***P. aeruginosa***	12.13 %	13.00 %	12.11 %	5.37 %	0.69 %
***E. coli***	0.00 %	6.83 %	6.12 %	3.42 %	4.83 %
***S. aureus***	33.98 %	26.00 %	33.20 %	20.21 %	6.31 %
***C. albicans***	8.44 %	28.73 %	33.75 %	24.78 %	20.83 %

**Table 4.** ∑FIC index for tramadol hydrochloride, fentanyl citrate and dexmedetomidine hydrochloride combinations against standard strains.

∑FIC	TH+FC	TH+DH	FC + DH	TH+FC+DH
***C. albicans***	1.17	0.58	1.53	0.45
***E. coli***	1.46	0.64	0.23	0.29
***S. aureus***	1.17	0.90	1.34	0.89
***K. pneumoniae***	0.72	0.90	3.23	0.53
***P. aeruginosa***	1.69	1.60	0.43	0.74

* Tramadol hydrochloride (TH), fentanyl citrate (FC) and dexmedetomidine hydrochloride (DH)
